# Polymorphisme de l'apolipoprotéine E dans la population du nord du Maroc: fréquence et influence sur les paramètres lipidiques

**DOI:** 10.11604/pamj.2013.15.157.2320

**Published:** 2013-08-31

**Authors:** Fatiha Benyahya, Amina Barakat, Naima Ghailani, Mohcine Bennani

**Affiliations:** 1 Institut pasteur du Maroc à Tanger. Tanger. Maroc; 2 Laboratoire de Génomique Humain, Faculté des Sciences et Techniques de Tanger. Tanger, Maroc

**Keywords:** Paramètres lipidiques, Maladies cardiovasculaires, Polymorphisme génétique, Gène APO E, lipid parameters, cardiovascular disease, genetic polymorphism, APO E gene

## Abstract

**Introduction:**

L'objectif de ce travail est de déterminer les fréquences alléliques et génotypiques des sites polymorphes situés dans le gène de l'apolipoprotéine E (apo E) ainsi que leur impact sur les paramètres cliniques et lipidiques dans un échantillon de la population du nord du Maroc cliniquement diagnostiqué ADH.

**Méthodes:**

Le génotype de l'apo E a été analysé par séquençage direct chez 46 patients cliniquement diagnostiqués ADH selon les critères standards.

**Résultats:**

Les fréquences des allèles epsilon 3, epsilon 2 et epsilon 4 ont été respectivement 78.3%, 2.2% et 19.6%. La fréquence de l'allèle epsilon 4 est très élevée chez la population du nord du Maroc en comparaison avec les populations des autres régions marocaines. Elle est similaire à celle rapportée dans les pays de l'Europe du nord. Les taux du cholestérol total, du cholestérol LDL ainsi que la présence des xanthomes et les maladies cardiovasculaires ne différent pas entre les génotypes de l'apoE. En revanche, les résultats ont montré une influence de l'allèle epsilon4 sur le taux des triglycérides chez les sujets obèses.

**Conclusion:**

Le génotype de l'apoE ne peut expliquer le phénotype clinique et biochimique présenté par des patients du Nord du Maroc cliniquement diagnostiqués ADH.

## Introduction

L'apoE est une composante majeure prenant part dans le processus général d'homéostasie du cholestérol. Son rôle dans le métabolisme lipidique fait l'objet de travaux de plusieurs groupes qui révèle la capacité qu'avait l'apoE de diriger la destinée métabolique des lipoprotéines. Le polymorphisme des isoformes E2, E3 et E4 interviennent au niveau du site d′interaction avec les récepteurs LDL et entraînent des changements de charges. Ces changements pourront modifier les interactions avec les récepteurs. L′isoforme E2 serait moins affine que les isoformes E3 et E4 pour ces récepteurs, l′isoforme E4 étant elle plus affine que E3. La présence de l'isoforme E4 se traduit par un métabolisme accéléré des lipoprotéines riches en triglycérides. Bien que son effet soit indétectable à l'échelle individuelle, le polymorphisme génétique de l'apoE determinerait 10% de la variance des taux de cholestérol dans les populations. Ce polymorphisme intervient ainsi dans la variation de la prévalence des maladies cardiovasculaires [1], en participant à la clairance du cholestérol médiée par des récepteurs [2] et en influençant la clairance des lipoprotéines riches en triglycérides [3]. Chez les sujets porteurs de l′allèle epsilon 4, le risque de maladies cardiovasculaires est plus élevé [4]. Par ailleurs, l′allèle epsilon4 est associé à la forme précoce de la maladie d′Alzheimer [5]. Les bases moléculaires de ce polymorphisme sont des substitutions dans les codons 112 et 158 de l′exon 4 du gène de l′apo E. Trois isoformes majeures résultent de ce polymorphisme: E2, E3 et E4 [6].

L′effet de génotype de l′apo E sur des concentrations lipidiques [7–20] et le risque coronaire [8, 9, 12, 14, 19] chez les sujets dislipémiques a été précédemment étudié avec des résultats variables [21]. A notre connaissance, aucune étude n'est réalisée au nord du Maroc pour déterminer la fréquence des allèles de l'apoE et leur effet sur le profile lipidique de cette population. D'autre part, les études menées dans des populations d'autres régions du Maroc (rabat et régions) ont concernés des sujets saints. Pour clarifier la contribution des génotypes de l'apoE dans le profile lipidique et MCV chez des patients dislipémiques, nous avons analysé le génotype de l′apo E dans un échantillon des probands du nord du Maroc qui ont un phénotype clinique et biochimique d'ADH.

## Méthodes

### Population étudiée

Notre population a été sélectionnée à l′aide du questionnaire établi pour le diagnostique clinique des patients des ADH chez la population du nord du Maroc. Ce questionnaire tient en compte les éléments suivants: LDLc > 2g/l, CT > 2.5 g/l, statut familial, niveau d′éducation, fumeur/non-fumeur, antécédents cardiovasculaires, tension artérielle, prise de médicaments, âge, poids et sexe. Les sujets présentant une glycémie supérieure à 1.1 g/l ont été exclus de cette étude.

La population étudiée comprend 46 sujets (27 femmes et 19 hommes) avec une moyenne d′âge 57 ± 10 ans, se rendant au centre de biologie médicale de l'Institut Pasteur du Maroc à Tanger pour un bilan lipidique. Tous les sujets sont originaires du nord du Maroc. L′âge, le poids, la tension artérielle, la taille sont enregistrés, l′indice de masse corporelle (BMI) est calculé et les paramètres lipidiques sont dosés.

### Analyse biochimique

Le sang veineux est prélevé après un jeûne d′au moins 10 heures. 10 ml sont recueillis sur EDTA pour l′étude moléculaire et 5 ml sont recueillis sur un tube sec pour le dosage des paramètres lipidiques. La glycémie, le cholestérol total et les triglycérides sont dosés par la méthode enzymatique standard sur l′automate (Dade Behring). Le dosage de l'apo B est réalisé par enzymo-immunologie sur le même automate.

### Analyse génétique

Pour le génotypage de l′apo E, l′ADN est extrait à partir des leucocytes selon la technique de Miller [22], et un séquençage direct a été réalisé pour explorer l′exon 4 du gène de l′apo E en utilisant l'analyseur génétique 3730^®^ (ABI), les amorces sont désignés par le logiciel primer3 [23] et sont synthétisés par Sigma. La détermination du génotype de l′apo E est réalisée en utilisant le logiciel de génotypage SeqScan, version 3.1, ABI.

### Analyses statistiques

Les moyennes et les écartypes ont été calculés par calcul direct sur excel (office 2007). Le programme ANOVA du logiciel STATISTICA version 8 a été utilisé pour évaluer les différences de paramètres cliniques et biologiques entre les différents groupes de patients.

## Résultats

La population de notre étude est composée de 46 patients qui présentent un phénotype clinique et biologique de l'ADH défini selon MEDPED.

La distribution et la fréquence des allèles du polymorphisme de l'apoE selon le sexe dans la population étudiée sont montrées sur le Tableau 1. Aucun sujet ne porte les génotypes E2/E2 et E2/E4, un seul sujet porte le génotype E2/E3 (2.2%), 8 sujets sont hétérozygotes pour le génotype E3/E4 (17.3%) et un seul patient homozygote pour le génotype E4/E4 (2.2%). Les fréquences des allèles ε2 (E2/E3), ε3 (E3/E3), et ε4 (E4/E4 et E3/E4) chez notre population sont de 2.2%, 78.3% et 19.6% respectivement. Nos résultats montrent que l'allèle ε4 est lié au sexe masculin chez le groupe de patients étudiés. La fréquence de l'allèle ε4 de l′apo E chez la population du nord du Maroc atteint 19.6%. Elle diffère de celle rapportée précédemment pour les populations d'autres régions du Maroc [24–26]. Elle est similaire à celle rapportée pour les populations de l′Europe du nord qui détiennent la fréquence la plus élevée de l′allèle epsilon4. Elle atteint 20% en Suède [27], 22% en Irlande du Nord [28] et 24% en Finlande [29] (Tableau 2).

**Tableau 1:** Distribution et fréquence des allèles des polymorphismes de l'apoEselon le sexe chez la population du Nord du Maroc

	Population Total n (%)	Femmes n (%)	Hommes n (%)
**Génotypes**			
E2/E2	0	0	0
E2/E3	1(2.2)	1 (3.7)	0
E3/E3	36 (78.3)	26 (96.3)	10 (52.6)
E3/E4	8 (17.3)	0	8 (42.1)
E4/E4	1(2.2)	0	1 (5.3)
E2/E4	0	0	0
**Fréquences des allèles**			
ε2	1(2.2)	1 (3.7)	0
ε3	36(78.3)	26 (96.3)	10 (52.6)
ε4	9(19.6)	0	9 (19.6)

ε4 comprend les génotypes E3/E4 et E4/E4 ; ε2 comprend le génotype E2/E3

**Tableau 2:** Fréquences alléliques dans la population du nord du Maroc comparées à d'autres groupes ethniques

Population étudiée	ε4	ε3	ε2
Nord du Maroc *	19.6	78.3	2.2
Maroc [24]	11	84	5
[25]	10	86	4
[26]	15.7	79.8	4.5
Tunisie [31]	8.1	84.6	7.3
Algérie [32]	10	84	6
Espagne[35]	10	85	4
[21]	9	89	2
France [33]	12	77	8
[34]	8	72	8
Suède [27]	20	72	8
Irlande du Nord [28]	22	66	12
Finlande [29]	24	70	6

* Population étudiée

Les résultats de notre étude génétique précédente réalisée sur les 46 patients ont montré que 5 patients portent des mutations sur le gène RLDL [30]. Les 5 patients sont homozygotes pour l'allèle ε3 de l'apoE ce qui exclu l'influence des allèles de l'apo E chez les patients hétérozygotes HF de notre population. Les 41patients restant ne portent aucune mutation ni sur le gène *RLDL* ni sur le gène *APOB* ou le gène *PCSK9* [30]. La contribution de génotype de l'apoE dans le métabolisme des lipides et le risque des maladies cardiovasculaire est étudié précédent [19, 20]. Pour clarifier la contribution des génotypes de l'apoE dans le profile lipidique et clinique (maladies cardiovasculaires et xanthomes) chez ces 41 patients, nous avons analysé de génotype de l'apoE.

Pour analyser l′influence du génotype de l′apo E sur les paramètres lipidiques et les caractères cliniques chez 41 patients (24 femmes et 17 hommes), nous avons regroupé les sujets en ceux qui portent l'allèle ε2, ceux qui portent l'allèle ε3 et ceux qui portent l'allèle ε4. Sous réserve du faible nombre de l′allèle ε2 (1 seul patε4 (E4/E4 et E4/E3) (Tableau 3). Nous remarquons ainsi qu'il n'y a pas de différence significative entre les 2 allèles de l'apoE pour l'âge, la tension artérielle (TA), le cholestérol total, le cholestérol LDL. Le taux des TG augmente significativement avec l'allèle ε4 (génotypes E4/E4 et E4/E3) (p < 0.05).

**Tableau 3:** Influence des allèles de l'apoE sur les données physiologiques et cliniques et les paramètres lipidiques

Paramètres	ε3 (E3/E3)	ε4 (E4/E3+ E4/E4)
Nombre de sujets	31	9
Age (années)	58±7	59±7
BMI (kg/m^2^)	26±5	30±2*
TA (mm Hg)	15±3	14±3
Cholestérol total (mg/dl)	278	300±15
LDL cholestérol (mg/dl)	219± 27	230±25
Triglycérides (mg/dl)	120±12*	210±18*
Apo B (g/l)	135±15*	148±20*
t.Xt (%)	17	20
MCV (%)	18	20

* *P*<0.05

Les taux des TG et de l'apoB sont significativement augmentés chez les sujets porteurs de l'allèleε4 en comparaison avec les porteurs de l'allèle ε3 (p < 0.05). La fréquence des maladies cardiovasculaires (MCV) ne diffère pas entre les sujets porteurs de l'allèle ε4 et ceux porteur de l'allèle ε3. Contrairement à l'étude marocaine précédente [24], nos résultats ont montré que le génotype de l′Apo E a une association avec le BMI.

Comme a été signalé précédemment, aucune femme de groupe de patients étudiés ne porte l'allèle ε4. La comparaison des données physiologiques et les paramètres lipidiques chez les femmes et les hommes porteurs de l'allèle ε3 avec les hommes porteurs de l'allèle ε4 (E4/E4 et E4/E3) a montré que les femmes porteuses de l'allèle ε3 ont un BMI (28kg/m^2^) plus élevés que les hommes du même allèle (26kg/m^2^) mais il est comparable au BMI des hommes de l'allèle ε4 (30 kg/m^2^). Les hommes de l'allèle ε4 ont des concentrations de triglycérides significativement plus élevées que les hommes et les femmes de l'allèle ε3 (p< 0,05). Le taux de triglycérides est augmenté chez les patients porteurs de l'allèle ε4 mais cette augmentation n'est pas significative statistiquement.

## Discussion

Il est bien établi que la fréquence des allèles de l′apo E diffère selon l′origine ethnique des populations étudiées. La fréquence de l′allèle epsilon4 est un facteur de risque pour la forme précoce de la maladie d′Alzheimer, ainsi que pour l′hypercholestérolémie et la prédisposition aux maladies cardiovasculaires qui en découlent [1].

Notre travail est le premier au Maroc qui a analysé l'influence de génotype de l'apoE chez des sujets hypercholestérolémiques [24-26] et c'est le premier travail qui a déterminé la fréquence des allèles de l'apoE dans la population du nord du Maroc.

La distribution des allèles de l′apo E dans notre série diffère de celle rapportée dans des populations d'autres régions marocaines [24-26] mais elle est conforme avec les distributions des allèles de l'apoE identifiés dans d′autres populations. En effet, l′allèle epsilon3 est l′isoforme majoritaire dans toutes les populations, puisque sa fréquence est de plus de 70% alors que l′allèle epsilon2 est le moins fréquent (Tableau 2).

Le résultat important de notre étude est la fréquence remarquablement élevée de l'allèle ε4 (19.6%) chez la population du nord de Maroc. La fréquence de l′allèle epsilon4 chez la population du nord de Maroc se rapproche de celle de l′Europe du nord (Suède [27] Irlande du nord [28] et Finlande [29]. En revanche, des différences significatives existent entre nos résultats et ceux des autres populations marocaines [25-27], de la population maghrébine (Tunisie [31] et l'Algérie [32]) et des populations de l'Europe de sud (France [33, 34], Espagne [21, 35]) (Tableau 2). Une étude Espagnole précédente a comparé les fréquences des allèles de l'apoE dans les populations de l'Afrique du nord et dans la population de la péninsule ibérique et a montré que la fréquence de l'allèle ε4 augmente en se dirigeant du sud vers le nord de l'Afrique [36]. Cette différence est probablement due à la différence de tailles des échantillons étudiés. Nous avons réalisé l'étude sur 41 patients alors que les autres études ont analysés des échantillons de grandes tailles (100 sujets [24] et 140 sujets [26]. Une autre explication c'est que nous avons travaillé sur des patients hypercholestérolémiques avec un profile clinique et biochimique d'ADH alors que les autres auteurs ont étudié des sujets sains.

Cette différence pourrait aussi être expliquée par la différence d'origines ethniques des populations analysées. En effet, les études précédentes réalisées chez des populations marocaines ont montré que la distribution des allèles de l'apoE différent entre la population d'origine berbère [26] et la population de Rabat [24]. D'autre part, le mode de vie économique peut influencer la distribution des allèles de l'apo E dans les populations. En effet, l′approvisionnement en nourriture était jusqu′au passé récent faible et difficilement disponible dans la région du nord du Maroc, les résultats de l'étude de *Corbo* et *Scacchi* [37] ont montré que la fréquence de l'allèle ε4 de l'apoE augmente chez les populations ayant un tel mode de vie économique.

Le deuxième résultat important de notre étude est que les génotypes de l'apo E n'ont pas d'influence ni sur les paramètres lipidiques (CT et LDLc), ni sur les données cliniques (CVD et xanthomes) chez les sujets dislipémiques. Il a été suggéré que le génotype de l'apoE ne contribue pas significativement dans le risqué des MCV [21]. Une étude espagnole a montré, chez des adultes avec hypercholestérolémie familiale présentant un risque élevé de maladies cardiovasculaires, que le génotype de l'apoE n'est pas associé aux MCV ou aux taux des lipides contrairement à l'effet établi chez la population générale [21]. En outre, l'âge moyen de groupe de patients concernés par notre étude est de 57 ans. Une étude comparative des paramètres lipidiques et d'infarction myocardique chez des sujets d'âge >35 ans a montré que la variation de ces paramètres est due à l'influence de l'âge et non pas au génotype de l'apoE [38, 39]. En revanche, nos résultats ont montré l'influence du génotype de l'apoE sur la concentration des triglycérides qui est significativement élevée chez les sujets porteur de l′allèle ε4 en comparaison avec les sujets porteurs de l'allèle ε3. Nos résultats ont montré que ces sujets sont obèses avec une moyenne de BMI de 30 kg/m^2^. Ce résultat confirme le travail de Fumeron et al [40] qui a rapporté une association remarquable de l'allèleε4 avec l'hypertriglycéridemie chez la population obèse en France. Gueguen et al. [41] a aussi montré que les individus porteurs de l'allèle ε4 avaient une importante élévation de taux de triglycérides en comparaison avec les sujets porteurs de l'allèle ε3 [27]. L'augmentation de BMI chez les femmes porteuses de l'allèle ε3 par rapport aux hommes porteurs de l'allèle ε3 peut être due à l'âge. L'âge moyen des femmes étudiées est 57 ans; depuis l′âge moyen 52 ans, beaucoup d′entre les femmes pourraient être ménopausées. Ainsi, beaucoup de femmes pourraient avoir l′obésité d′androïde [31].

## Conclusion

Notre travail est le premier au Maroc qui a analysé l'influence de génotype de l'apoE chez des sujets dislipémiques et c'est le premier travail qui a déterminé la distribution des allèles de l'apoE dans la population du nord du Maroc. Un résultat important de notre étude est la fréquence très élevée de l'allèle ε4 chez la population du nord du Maroc ce qui confirme la différence de l'origine ethnique de cette population par rapport aux populations des autres régions du Maroc. Un deuxième résultat important c'est que le génotype de l'apoE n'est associé ni au profile lipidique ni au profile clinique présenté par les patients cliniquement diagnostiqués ADH au nord du Maroc. L'interaction entre le polymorphisme de l'apolipoprotéine E, l'obésité et le changement de taux des triglycérides dans notre population confirme les études épidémiologiques annonçant que l′allèle ‘4 augmente le risque de l'hypertriglycéridemie parmi des individus obèses Le génotype de l'apoE ne peut expliquer le phénotype clinique et biochimique présenté par des patients cliniquement diagnostiqués ADH. Plus d′études devraient être effectuées pour chercher d′autres gènes responsables de l'ADH chez la population du nord du Maroc.
